# Algorithmic management in the global gig economy: an interdisciplinary systematic literature review and critical discourse analysis

**DOI:** 10.3389/fsoc.2026.1743445

**Published:** 2026-04-15

**Authors:** Bianca Ifeoma Chigbu

**Affiliations:** Faculty of Law, Social Sciences & Humanities (Department of Social Sciences), Walter Sisulu University, Mthatha, South Africa

**Keywords:** algorithmic control, algorithmic management, digital labor, gig economy, global labor, platform economy, platform work, work regulation

## Abstract

Algorithmic management—the delegation of managerial functions to algorithmic systems has become a defining feature of work in the global gig and platform economy. This article presents a systematic literature review (SLR) of 103 scholarly sources, complemented by a targeted critical discourse analysis (CDA) to examine how algorithmic management operates across diverse regions and is discursively framed. While gig work engages millions of workers worldwide, scholarly attention has centered disproportionately on Western contexts. I develop an interdisciplinary research framework that integrates sociology, labor studies, digital economy, and law to analyze the implications of algorithmic management for labor practices, worker autonomy, and regulation. The study asks: (1) what dominant themes and findings emerge in the literature across regions and contexts; (2) how these themes are discursively framed in scholarly and policy texts; and (3) what research gaps and policy implications emerge from this synthesis. The SLR reveals several prevalent themes: the tension between promised flexibility and algorithmic control, worker resistance and agency, biases and fairness in algorithmic systems, and the influence of local institutional contexts, while the CDA uncovers how academic narratives often oscillate between portraying algorithmic management as an extension of digital Taylorism and a catalyst for novel forms of worker autonomy. This review offers contributions by mapping the state of knowledge, highlighting a critical gap in global and comparative analysis, and proposing an agenda for future research and policymaking.

## 1 Introduction

Digital labor platforms such as Uber, Bolt, Rideshare, Upwork, and Deliveroo have radically transformed the organization of work worldwide. Over the past decade, the *gig economy*, characterized by short-term, on-demand work mediated through online platforms (De Stefano, 2016a; ILO, 2021a; Wood et al., 2019a), has grown exponentially, engaging millions of workers worldwide in service provision across transportation, delivery, care work, and other sectors. In 2023, it was estimated that online on-demand labor could account for up to 12% of the global labor force (World Bank, 2023), underscoring the worldwide scale and significance of this trend. A core driver of this new work model is *algorithmic management*, where computational algorithms assume traditional managerial functions in hiring, task assignment, performance monitoring, and even discipline (Duggan et al., 2020; Kellogg et al., 2020; Möhlmann et al., 2021; Rosenblat, 2016). Gig platforms champion this algorithmic approach to achieve unprecedented efficiency and flexibility, ostensibly allowing workers to choose when, where, and how much to work (Dubal, 2020; Duggan et al., 2020; Möhlmann and Zalmanson, 2017; Rosenblat, 2018; Vallas and Schor, 2020). However, emerging evidence reveals a far more complex reality: algorithmic management often reproduces and intensifies managerial control, raising urgent questions about worker autonomy, fairness, and wellbeing in platform-mediated work (Christin, 2020; Duggan et al., 2020; Gong, 2025; Griesbach et al., 2019; HRW, 2025; Kellogg et al., 2020; Rosenblat and Stark, 2016; Wood et al., 2019a; Zhang M. M. et al., 2025; Winner, 2017).

Scholars across disciplines have begun to scrutinize the double-edged nature of algorithmic management in the gig economy. On one hand, algorithms optimize resource allocation and provide consumers instant access to labor, contributing to seamless user experiences and new income opportunities for workers (Li et al., 2025; Möhlmann et al., 2025; OECD, 2023). On the other hand, the data-driven, automated control systems employed by platforms can subject workers to relentless surveillance, opaque decision-making processes, and precarious working conditions (Anwar and Graham, 2021; Duggan et al., 2023; HRW, 2025; Rahman, 2021; Wood et al., 2018). For example, ride-hailing and delivery drivers are managed through continual GPS tracking, customer ratings, automated performance alerts, and dynamic pricing mechanisms that prioritize efficiency over worker wellbeing (HRW, 2025; Li et al., 2025; Möhlmann et al., 2022; Wood et al., 2018). High-profile incidents, such as accidents attributed to drivers rushing to meet unrealistic algorithmic deadlines, have spotlighted the human costs of these systems (Shannon et al., 2024). Consequently, a growing body of research highlights issues including loss of autonomy, intensified work pressure, job insecurity, and power asymmetries between workers and platforms in an algorithmically managed workplace (Dong et al., 2025; Griesbach et al., 2019; Lang et al., 2023; van Zoonen et al., 2025).

Crucially, current scholarship suggests that algorithmic management is not a monolithic phenomenon; its implementation and impact vary across different global regions and socio-regulatory contexts. Early studies and influential books (Gray and Suri, 2019; Rosenblat, 2018) focused mainly on North America and Europe, emphasizing themes of “algorithmic despotism” and the emergence of a “new global underclass” of gig workers. However, gig work is equally prevalent in Asia, Latin America, and Africa, often under different labor market conditions and legal regimes. Nevertheless, these contexts remain underrepresented in the literature. This highlights a novel and impactful research gap: a need for comparative, globally inclusive analyses of how algorithmic management operates and is experienced in diverse regions. As many scholars note, platform workers' experiences can vary significantly based on the configuration of algorithmic systems, local social norms, and labor history (Anwar and Graham, 2021; Cameron et al., 2021; Kenney and Zysman, 2020; van Doorn, 2017; Wood et al., 2019a; Zhu J. et al., 2024). To date, few studies have explicitly contrasted these cross-regional differences or examined how local legal and cultural factors mediate algorithmic management practices.

Another gap in existing research lies in the discursive construction of algorithmic management. Much of the literature adopts a critical stance, invoking metaphors of “*digital Taylorism*” (Moore, 2017; Park and Ryoo, 2023) and “*electronic panopticons*” (Newlands, 2021; Rosenblat and Stark, 2016; Woodcock, 2020) to characterize the control strategies employed by gig platforms. This critical discourse, rooted in sociology and labor process theory, frames platform algorithms as instruments of exploitation, casting gig workers as precarious laborers lacking voice or rights (Huws, 2019; Prassl, 2018; van Doorn, 2017; Wood et al., 2019a). In contrast, a managerial or techno-optimist discourse more common in information systems and management fields may emphasize innovation, “*algorithmic HRM* (human resource management)” efficiencies, and data-driven optimization, sometimes echoing platform narratives of worker empowerment and entrepreneurship (e.g., Cameron et al., 2024; Cui et al., 2024; Keegan and Meijerink, 2025). These differing frames have not been systematically analyzed. Understanding the dominant narratives and rhetoric in scholarly and public debates is crucial because discourse influences policy agendas and shapes the public perception of the gig economy. By employing elements of CDA, this study aims to illuminate how language and framing in the literature reflect deeper power dynamics and assumptions about gig work (e.g., freedom vs. precarity, innovation vs. exploitation), and how these discourses may influence real-world outcomes.

In response to these gaps, this article conducts a SLR of academic research on algorithmic management in the gig/platform economy, complemented by a CDA of selected scholarly and policy texts. The SLR is used to identify the dominant empirical and conceptual themes in the literature, while the CDA is used to interpret how those themes are discursively constructed, legitimized, and contested. I integrate perspectives from sociology, organizational studies, human resource management, information systems, labor law, and ethics to build a comprehensive understanding and to formulate an interdisciplinary research framework. In doing so, I addressed the following research questions:

- *RQ1: What are the prevalent themes and findings in scholarly literature on algorithmic management in the gig/platform economy, and how do these findings differ across global regions and contexts?*
- *RQ2: How are the dominant themes identified in the SLR discursively framed in scholarly and policy texts?*
- *RQ3: What research gaps and policy implications emerge from this synthesis?*

By answering these questions, this review offers several contributions. First, it provides the most up-to-date synthesis of an increasingly prolific research area, cataloging insights from 103 included studies (drawn from 314 records identified across databases, 2010–2025). I presented a structured overview of key concepts (with definitions), theoretical approaches, empirical findings, and regional emphases, aided by summary tables and figures for clarity. Second, through CDA, I added a reflexive layer of analysis, revealing how the academic conversation itself may reinforce or challenge power structures—for example, by either perpetuating a narrative of inevitability around algorithmic control or by giving voice to worker agency and alternatives. Third, I proposed a conceptual framework that bridges interdisciplinary viewpoints and highlights points of convergence (e.g., consensus on the need for worker protections) and divergence (e.g., how different fields value efficiency vs. equity). Finally, I discussed the implications for practice and policy, including the urgent need to update labor regulations (e.g., clarifying employment status and rights of gig workers), to ensure algorithmic transparency and accountability, and to promote ethical AI management practices that uphold human dignity.

The remainder of this article is organized as follows. I begin with the conceptual framework, explaining how interdisciplinary theories inform our perspective. The methodology section then details the systematic review protocol and discourse analytic approach. I proceed to the findings, where I report descriptive trends and thematic insights from the SLR, followed by the CDA results on discursive patterns. In the discussion, I interpret these findings, highlight the identified research gap and its significance, and elaborate on the legal, ethical, social, and policy implications. I also outline a future research agenda addressing the gaps. Finally, the conclusion summarizes the contributions and calls for a more inclusive, fair, and critically informed approach to studying and governing algorithmic management in the global platform economy.

## 2 Conceptual framework: integrating interdisciplinary perspectives

Studying algorithmic management in the global gig economy necessitates an interdisciplinary conceptual framework due to the phenomenon's complex socio-technical nature. In this section, I outlined the theoretical lenses and concepts that guide my analysis, drawing from sociology, organizational studies, HRM, critical management studies, and legal scholarship. By integrating these perspectives, I aimed to create a holistic framework that accommodates both the *material practices* of algorithmic management and the *discursive constructions* that surround them.

**Figure 1:**
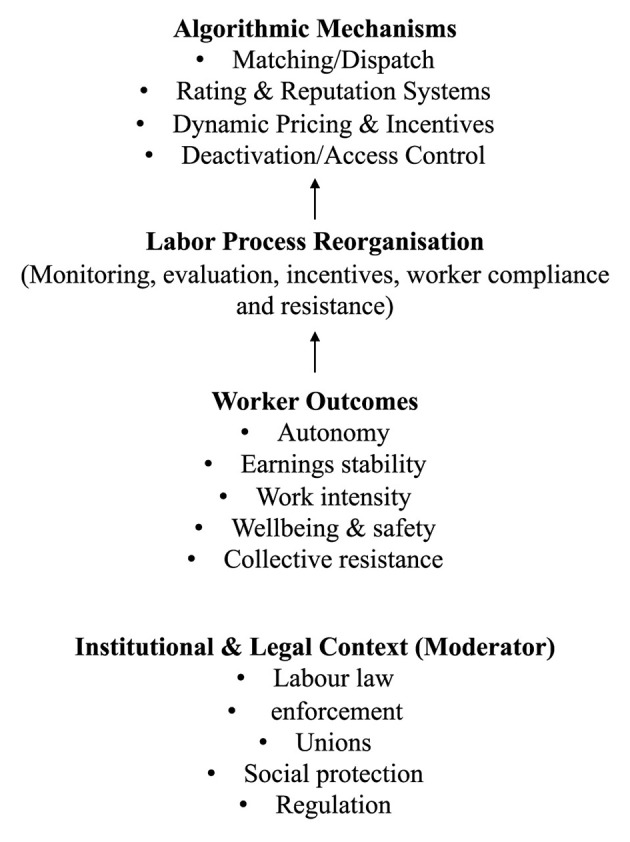
Conceptual model of algorithmic management and worker outcomes.

Figure 1 presents the simplified conceptual model that guided the review, illustrating how algorithmic management mechanisms shape worker outcomes within the gig economy, while institutional and legal conditions moderate these relationships. This review uses a streamlined conceptual framework built around three complementary lenses: (1) Labor Process and Power (Braverman, 1998); (2) Algorithmic Control Systems (Moore and Joyce, 2020; Veen et al., 2020; Wood et al., 2019a); and (3) Institutional and Legal Mediation. These lenses specified how platform algorithms enact managerial control, how workers respond, and how outcomes vary across regulatory and institutional settings. *Labor Process and Power*—algorithmic management is treated as a contemporary control regime that reorganizes the labor process through digitally mediated monitoring, evaluation, and incentive structures. This lens foregrounds asymmetries of power, mechanisms of consent and resistance, and the everyday tactics workers use to navigate or contest algorithmic control. *Algorithmic Control Systems*—I conceptualized platforms as socio-technical control systems in which specific algorithmic mechanisms translate organizational objectives into rules that shape work allocation and behavior. Across the reviewed literature, four recurrent mechanism types appear: (a) matching/dispatch and task allocation; (b) rating and reputational scoring; (c) dynamic pricing and incentive/bonus logic; and (d) deactivation and access-control logic (including automated warnings, suspensions, and removal). These mechanisms interact to produce observable outcomes such as autonomy constraints, income volatility, intensified pace, safety pressures, and psychosocial strain. *Institutional and Legal Mediation*—the same algorithmic mechanisms can yield different worker outcomes depending on institutional context, including employment classification, enforcement capacity, collective representation, and platform-specific regulation. This lens treats legal and institutional conditions as moderators that shape both the design of algorithmic controls and the degree of voice and contestation available to workers.

## 3 Methodology

### 3.1 SLR methodology

To address RQ1 and RQ3, I conducted a SLR following rigorous and transparent procedures. I adhered to guidelines inspired by the PRISMA framework for systematic reviews, aiming to ensure a comprehensive and unbiased collection of relevant literature. The SLR process involved literature search, study screening with inclusion/exclusion criteria, data extraction, and synthesis.

#### 3.1.1 Literature search

I performed an extensive search across multiple academic databases and sources to capture the interdisciplinary nature of the topic. Specifically, I searched in EBSCOhost, Web of Science, Scopus, and Google Scholar. The search terms combined keywords related to algorithmic management and the gig/platform economy. For example, my queries included “gig econom^*^” OR “platform work” OR “app-based work” OR “on-demand work” OR “algorithmic manag^*^” OR “algorithmic control” OR “worker surveill^*^” OR “AI manag^*^” OR “Uber” OR “Bolt” OR “Deliveroo” “Rideshare” AND “Digital Labor Rights” OR “fairwork” OR “gig workers' rights” OR “digital labor” OR “gig work AND Africa” OR “platform AND surveillance,” etc. I also employed snowballing techniques (checking the references of key papers) to identify additional sources. I limited the search primarily to literature published between 2010 and 2025, as algorithmic management in gig work is a relatively recent topic that gained traction in the mid-2010s. However, I included earlier theoretical works to ensure theoretical saturation.

**Figure 2:**
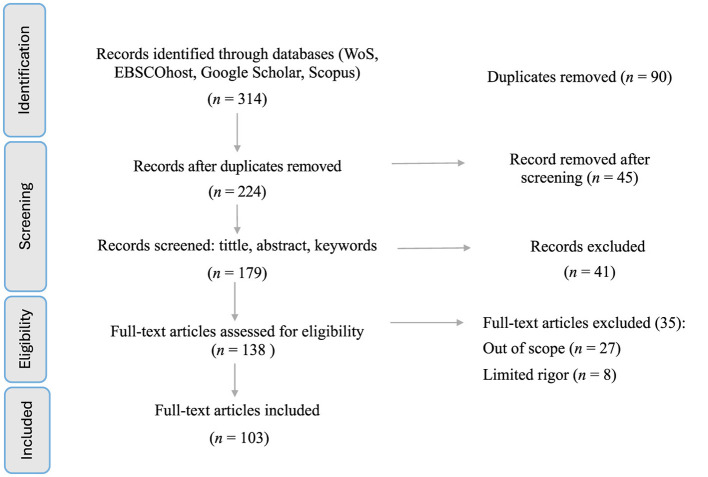
PRISMA flow diagram (N = 103).

To strengthen methodological transparency and replicability, Table 1 summarizes the SLR search protocol, including database coverage, search dates, fields searched, filters applied, and the deduplication and screening procedures. This protocol underpins the PRISMA flow (Figure 2) and the final included corpus (*n* = 103). Screening and eligibility assessment were conducted by a single reviewer (the author). While dual screening can reduce selection bias, single-reviewer screening is a recognized approach in solo-authored reviews when resources are constrained. To support consistency, I applied predefined inclusion/exclusion criteria uniformly across stages, documented reasons for full-text exclusions (summarized in Figure 2), and ensured that all descriptive results and synthesis refer only to the final included studies (*n* = 103).

**Table 1:** SLR protocol for replicability.

Component	Specification
Databases searched	Web of Science (WoS), Scopus, EBSCOhost, Google Scholar
Search dates	January–February 2025
Fields searched	Title, Abstract, and Keywords (database-dependent field filters applied where available)
Core Boolean search logic (adapted per database syntax)	(“algorithmic management” OR “algorithmic control” OR “AI management” OR “platform work” OR “gig economy” OR “app-based work” OR “on-demand work” OR “crowdwork”) AND (“surveillance” OR “monitoring” OR “worker rights” OR “labor regulation” OR “fairwork” OR “autonomy” OR “precarity”)
Time window	2010–2025
Language	English
Document types (included)	Peer-reviewed journal articles, conference papers, books/book chapters, and high-impact institutional reports
Deduplication	Duplicates removed using reference management software; duplicates were then checked manually for verification
Screening workflow	Title/abstract/keyword screening → full-text eligibility assessment → final inclusion (*n* = 103), aligned with PRISMA (Figure 2)
Quality control/appraisal	No formal quality scoring tool was applied; studies were excluded at the full-text stage when out of scope or where methodological rigor was insufficient (Figure 2)

The database search identified 314 records across Web of Science, EBSCOhost, Scopus, and Google Scholar (2010–2025). After removing duplicates (*n* = 90), 224 unique records remained. As shown in Figure 2, 45 records were removed prior to screening, 179 records were screened on title/abstract/keywords, and 138 full texts were assessed for eligibility; 35 full texts were excluded (27 out of scope; eight limited rigor), leaving 103 studies in the final analytical corpus.

#### 3.1.2 Study screening

From the initial pool, inclusion and exclusion criteria were applied to select studies most relevant to the research questions.

*Inclusion criteria:* (1) the study explicitly addresses algorithmic management/control in the context of gig or platform-based work (or closely related contexts like crowdwork, on-demand labor); (2) it provides empirical findings, theoretical insights, or substantial discussion on how algorithms manage labor, or on the effects/implications of such management; (3) published in English (given my language capacity), unfortunately, this meant excluding some non-English scholarship, which I acknowledge as a limitation; and (4) published in a credible outlet (peer-reviewed or by reputable institutions) to ensure quality. *Exclusion criteria:* I excluded studies that only tangentially mentioned gig platforms without focusing on management practices (e.g., an article about consumer behavior on Uber would be out of scope). I also excluded purely technical studies on algorithms that did not discuss human management implications (e.g., a computer science paper on routing optimization for deliveries, unless it discussed labor aspects). Screening proceeded in stages consistent with PRISMA (Figure 2): after deduplication (*n* = 224), 45 records were removed prior to screening, 179 records were screened on title/abstract/keywords, and 138 full texts were assessed for eligibility, yielding a final included corpus of 103 studies.

Full-text eligibility assessment was conducted on 138 articles. Thirty-five full texts were excluded at this stage (27 out of scope; eight limited rigor), resulting in a final corpus of 103 included studies for detailed data extraction and synthesis (Figure 2). The final corpus spans multiple disciplines, including sociology, management/HRM, information systems, law, labor economics, and regional studies.

I coded 103 studies to map methodological and regional patterns in the literature on algorithmic management (see Table 2). Each study was classified by author(s), year, title, research design, focal platform/sector, country/region of focus, findings, and implications/gaps noted. Although this study was coded during analysis, it was not included in the final summary table shown here because the simplified version reports only aggregated categories rather than individual entries. This enabled both quantitative and qualitative assessment of how the field has evolved and where its gaps remain.

**Table 2:** Research design distribution across 103 algorithmic management studies (2010–2025).

Research design	Number of studies
Qualitative/ethnographic/ interview-based/case study	Thirty-six studies rely primarily on interviews, ethnography, field observation, discourse analysis of worker forums, or in-depth case work.
Quantitative/survey/ statistical/econometric	Eighteen studies employ surveys, structural equation models, multilevel models, diary designs, crash statistics, difference-in-differences, and other methods.
Mixed-methods	Four papers combine qualitative interviews/ethnography with platform data, forum scraping, algorithm/market trace data, or quantitative outcome analysis, such as (Möhlmann et al. 2021) and (Waldkirch et al. 2021).
Conceptual/theoretical/ critical/review	Twenty-eight studies develop theory, define concepts, or synthesize literature without reporting original empirical fieldwork.
Policy/legal/regulatory/ institutional reports	13 are empirical, but their core aim is governance, labor rights, or regulation, not theory-building.

The distribution of research designs reveals a maturing yet uneven field. Qualitative and ethnographic studies dominate (36 of 103), reflecting the early focus on documenting lived experiences, control mechanisms, and worker resistance within gig platforms. Quantitative and econometric studies (18) emerged later, mainly in information systems and management disciplines, offering statistical evidence for patterns first observed qualitatively, such as burnout, engagement, and algorithmic pressure. Only four mixed-methods papers bridge experiential and structural levels, highlighting a persistent methodological gap. Conceptual and theoretical contributions (28) continue to shape key debates but remain largely Western-centric in nature. At the same time, 13 policy and legal studies mark growing institutional engagement from bodies such as the ILO, OECD, and EU.

Collectively, these patterns demonstrate that scholarship on algorithmic management is conceptually rich but methodologically and geographically uneven, substantial in theorization and qualitative depth, yet limited in quantitative integration and representation from the Global South.

**Figure 3:**
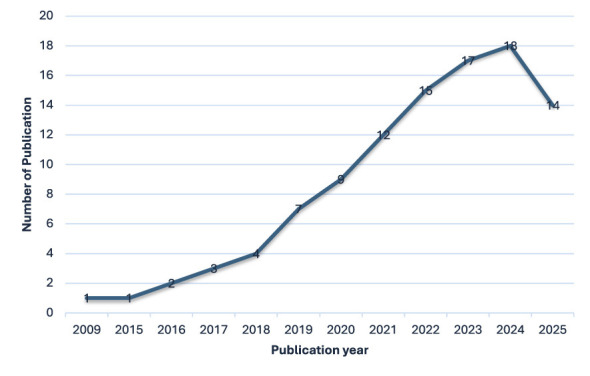
Temporal trend in publications on algorithmic management (2010–2025), based on the 103 studies included in the SLR. Publication counts reflect the number of included sources per year.

Figure 3 shows the temporal distribution of publications within the final review corpus. Counts are derived from the 103 studies included in the systematic literature review (2010–2025), with each study classified according to its publication year.

As shown in Figure 3, which reflects the distribution of the 103 included studies, a line graph with publication year on the *X*-axis (2010–2025) and the number of included publications on the *Y*-axis reveals a sparse research landscape before 2015, with only one or two publications appearing in isolated years. Scholarly attention began to accelerate around 2017–2018, followed by a pronounced surge between 2019 and 2023. The curve peaks between 2022 and 2024, when the annual number of publications reaches its highest level, reflecting the rapid institutionalization of “algorithmic management” as a core topic in digital-work research. Overall, more than two-thirds of all sources in the corpus were published from 2019 onwards, confirming that academic engagement with algorithmic management is a relatively recent and rapidly expanding field that parallels the global growth of the platform and gig economy.

**Figure 4:**
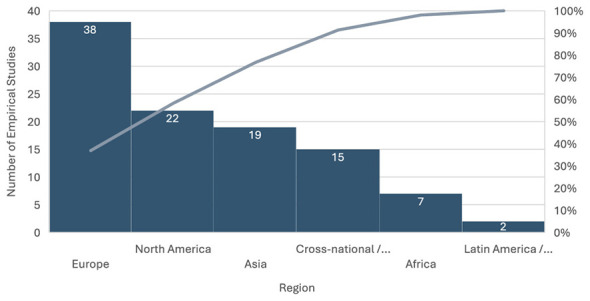
Geographical distribution of empirical studies.

I also compiled a chart of the geographical focus of empirical studies included in this study (Figure 4), which further illustrates the concentration of this work.

Approximately one-third of empirical research originates from Europe, with another fifth from North America and about one-fifth from Asia (dominated by studies in China, India, and other East-Asian contexts). In contrast, Africa and Latin America together account for fewer than 10 percent of empirical contributions. Approximately 15 percent of studies employ cross-national or global designs, often comparing multiple regions or examining global policy frameworks. This uneven distribution highlights a persistent geographic bias in the literature and underscores the need for a deeper empirical engagement with underrepresented regions, particularly Africa and Latin America.

#### 3.1.3 Data synthesis

Both qualitative synthesis (thematic analysis) and basic quantitative tallies were used to summarize findings. Thematic coding was applied to study findings to identify recurring themes such as “worker resistance strategies,” “legal challenges,” “algorithmic bias,” “health and safety issues,” “management strategies,” and “worker satisfaction.” I iteratively refined these themes. The CDA was then used to interpret how the dominant themes identified in the SLR were discursively constructed in selected scholarly and policy texts, including how different authors framed flexibility, control, precarity, and accountability. This integration linked substantive themes to discursive patterns. Overall, the SLR addressed RQ1 (what is known) and generated the thematic basis for RQ3, while the CDA provided the interpretive layer for RQ2.

### 3.2 CDA methodology

To tackle RQ2: examining how algorithmic management and gig work are discursively framed, I complemented the SLR with a CDA conducted on a purposive subset of texts drawn from the final review corpus (e.g., policy documents, media framing where relevant). The CDA drew from Fairclough's approach, which looks at text, discursive practice, and social practice. In practical terms, I focused on the language used by researchers and thought leaders in portraying algorithmic management and gig workers, aiming to uncover underlying assumptions, power relations, and potential biases in the discourse.

#### 3.2.1 Selection of texts for CDA

The CDA was conducted on a purposive subset of 35 focal texts selected from the 103 included studies, alongside 8 policy and institutional documents. Selection followed theoretical sampling logic, prioritizing disciplinary diversity, citation influence, and relevance to debates on algorithmic management and focusing on influential and representative texts across different disciplines. I chose the introductions and literature review sections of key academic papers (where authors often frame the problem and state their stance). For example, I closely read the introductions of (Duggan et al. 2020), Rosenblat's (2018) book preface, (Wood et al. 2019a), a law review article by (Dubal 2017a,b), and a management piece by (Kellogg et al. 2020), among others. Abstracts and conclusions are also insightful for distilled framing. Additionally, I looked at policy documents or op-eds by experts if available (e.g., an OECD policy paper's exec summary framing gig work as “opportunity vs. challenge”). I also included some media discourse for context, such as iconic magazine articles or public reports referenced in academic work (for example, an article by The Atlantic or The Guardian on gig workers, which academics cite to illustrate the public narrative).

#### 3.2.2 Analytical procedure

Textual coding was conducted using NVivo 14 and proceeded through two iterative coding cycles. The first cycle involved open coding of metaphors, evaluative language, and framing devices. The second cycle applied axial coding to group these elements into broader discursive categories, such as the use of terms like “flexibility” vs. “precarity,” “micro-entrepreneur” vs. “worker,” and “freedom” vs. “exploitation.” I cataloged how often and in what context such terms appeared. For instance, I noted that managerial literature usually uses relatively neutral or positive terms, such as “efficiency” and “innovation,” and frames algorithms as improving HR processes, whereas critical literature uses charged terms like “digital shackles” or “boss in the machine.” How are gig workers described? As “drivers,” “service providers,” “contractors,” “employees,” or “users of the app”? How are companies described? As “platforms,” “employers,” “intermediaries,” “marketplaces”? These choices indicate the author's perspective on responsibility and agency. For example, referring to Uber as a “client” of drivers vs. an “employer” is a significant discursive difference that aligns with either Uber's stance or labor's stance. I examined sentences to see if authors attribute outcomes to algorithms themselves (e.g., “the algorithm decides X”) or to the platform (e.g., “Uber enforces X through algorithms”). Anthropomorphizing the algorithm (“algorithms fire workers”) is a common trope that may downplay or highlight certain aspects. I analyzed these constructions. Words indicating judgment, such as “harmful,” “beneficial,” “controversial,” “fair/unfair,” “opaque,” “user-friendly,” etc., show the author's evaluation of algorithmic management. What metaphors or theories are invoked? e.g., references to “Taylorism,” “panopticon,” “consent” (Burawoy), or to neoliberalism and capitalist critique. These signal the ideological lens of the discourse community.

All coded segments were managed within NVivo 14 to ensure systematic organization, traceability of coding decisions, and transparency in the derivation of discursive patterns. Through this iterative coding process, three dominant discursive frames emerged inductively: (1) a techno-optimist frame emphasizing innovation and efficiency; (2) a critical-labor frame highlighting control, precarity, and exploitation; and (3) a legal-technical frame focusing on regulatory classification and governance. I found that a managerial/technocratic discourse prevalent in business/tech fields emphasizes efficiency, innovation, and sometimes assumes gig work is here to stay, focusing on optimization (how to make algorithmic management better and more acceptable). This discourse often suggests tweaks rather than questioning fundamentals (e.g., “with better algorithm design, we can improve worker satisfaction”). A critical discourse present in labor studies, sociology, and critical management, which frames algorithmic management as exploitative and an extension of capitalist control, often employing strong language (e.g., “a new form of exploitation in the digital age”). A legal/policy discourse which can vary, but often focuses on rights, classifications, and may use more formal language [e.g., “the dualism of legal identities” (Dubal, 2017b) or “worker reclassification”]. This discourse sometimes bridges—acknowledging flexibility but stressing the need for worker protection (some policy docs use a balanced frame of “opportunities and challenges”). In worker-centric narratives in interviews and ethnographies, sometimes the workers' own discourse is relayed (like drivers referring to “beating the algorithm” or calling it “the big boss”). Those voices in my corpus provided a ground-level discourse that was noted in the analysis.

I also considered silences and assumptions. CDA looks at what is not said. For example, does a discourse assume that gig work is primarily for young urban men and overlook women's experiences? Does it neglect global South contexts? I found that many mainstream discourses implicitly took a Western urban default. I highlight such gaps in my analysis as part of discourse critique.

Crucially, I examined how these discourses might influence or reflect power relations. For instance, a pro-platform discourse often found in tech industry white papers might frame gig workers as independent business owners. This framing justifies minimal labor obligations. Meanwhile, academic critical discourse may not penetrate policy if it is too removed or jargon-laden. By identifying this, we can discuss the practical implications of discourse, such as the need to reframe gig worker issues in a way that garners public support.

By combining SLR and CDA, the methodology does not treat the two components as parallel exercises. Rather, the SLR identifies the dominant themes in the literature, and the CDA interprets how those themes are framed, legitimized, or contested across selected texts. This integration provides both a structured synthesis of knowledge and a critical account of how that knowledge is constructed.

Finally, all findings from the SLR and CDA were synthesized to answer the RQs. The Findings section first presents the dominant themes emerging from the SLR and then shows how the CDA interprets those same themes discursively, highlighting the frames through which algorithmic management is understood and debated.

Throughout the methodology, I remained cognizant of ethical considerations, properly citing all sources, avoiding plagiarism by summarizing and synthesizing with attribution, analyzing the discourse of authors, and doing so respectfully and in context. The research did not involve human subjects directly (only published materials), so there were no human subject ethical issues, but I applied rigorous standards to represent others' work truthfully. I turn now to the presentation of the findings.

## 4 Findings

In this section, I present the study's findings in two linked parts. First, I summarize the key insights from the SLR, which is structured around major thematic areas that emerged from the 103 studies reviewed. I included descriptive statistics and tables to illustrate the research landscape (e.g., the distribution of studies by region and theme). Second, I use the CDA to interpret how these same themes are framed in scholarly and policy texts, and how those discursive patterns relate to power, legitimacy, and knowledge in the gig economy context.

### 4.1 SLR findings: thematic patterns in the literature

#### 4.1.1 Ubiquity of algorithmic control mechanisms

Across the reviewed literature, there is a consensus that algorithmic control is a ubiquitous and defining feature of gig work, manifesting in similar ways across platforms. Table 3 summarizes the typical components of algorithmic management in gig platforms as identified in various studies:

**Table 3:** Common components of algorithmic management in gig platforms, synthesized from recent empirical and theoretical literature.

Component of algorithmic management	Description/function	Example sources (studies)
Task allocation algorithms	Automated dispatch/matching of workers to tasks or customers, based on proximity, ratings, availability, etc.	Uber's dispatch algorithm (Rosenblat and Stark, 2016); Upwork job matching (Leicht-Deobald et al., 2022)
Monitoring and surveillance	Continuous tracking of worker location, actions, online status; logging of metrics (e.g., delivery times, response times).	China delivery study (Chen et al., 2025); (Rosenblat 2018) on Uber's GPS tracking
Performance evaluation metrics	Customer rating systems, acceptance rates, cancellation rates, on-time rates, etc., often aggregated into a performance score.	Uber 5-star rating system (Rosenblat, 2018); Food delivery on-time rate (Shannon et al., 2024)
Disciplinary algorithms	Automated enforcement of rules: e.g., temporary “time-outs” for low acceptance, algorithmic “deactivations” (terminations) for low ratings or violations.	Uber driver lockout after declines; Amazon MTurk account suspensions (Gray and Suri, 2019)
Dynamic pricing/payment systems	Algorithmically set pay rates (e.g., surge pricing, batch delivery pay formulas) and bonuses. Often opaque calculations.	Uber surge pricing (Kornberger et al., 2018); Instacart pay algorithm controversy
Gamification and nudges	Use of game-like elements (badges, levels) or behavioral nudges (“quests,” streak bonuses, forward dispatching rides before current ends) to encourage more work.	Lyft's bonus streaks (Scheiber, 2022); “Quests” in Uber (Vasudevan and Chan, 2022)
Communication via app interface	Workers communicate with the platform only through app interface or automated messages; human support is minimal or via scripted chatbots.	Driver quotes: “You can't call them…” (in McDaid et al., 2023); Lack of human manager contact (Veen et al., 2020).

The literature demonstrates that while exact implementations differ (e.g., Uber uses a 5-star rating, while Upwork uses client feedback and job success scores), these elements form a *toolkit of algorithmic management* that is consistently observed. This supports the notion that gig platforms have converged on a similar model of remote, automated labor management (Möhlmann and Zalmanson, 2017).

One interesting finding is that some platforms emphasize specific components more based on service type: for example, ride-hailing apps heavily utilize dynamic pricing (surge) to manage supply, whereas micro-task platforms prioritize worker reputation scores to match workers with future tasks. Yet, fundamentally, all rely on data-driven control. Several studies (e.g., Leicht-Deobald et al., 2022; Newlands, 2021) attempt to define “algorithmic management” by listing these components, reinforcing that the academic community largely agrees on what constitutes algorithmic management as a construct.

#### 4.1.2 Effects on worker autonomy and behavior

A strong theme is the tension between nominal autonomy and algorithmic control. Empirical studies consistently find that gig workers' autonomy is constrained in practice, as detailed in the literature review. In (Wood et al. 2019b)'s survey of UK and US workers, those in on-location gigs (ride-share, delivery) reported significantly less control over how they did their work and work pace compared to remote gig workers (online freelancing). This aligns with the qualitative evidence that constant monitoring and automatic penalizations push workers to conform to algorithmic expectations meticulously. For instance, an ethnographic study by (Rosenblat 2018) documented drivers feeling forced to accept rides to avoid punishment by the algorithm, effectively working “on command” of the app even though they are legally free to log off.

Multiple studies highlight changes in worker behavior due to algorithmic pressures, including “algorithmic improvisation,” where workers learn tricks to increase earnings within system rules, and conversely, risk-taking behavior such as speeding and traffic violations in delivery due to time pressure (Chen et al., 2025). A transportation study by (Lee et al. 2022) found a correlation between strict time countdowns and riders engaging in unsafe maneuvers—confirming quantitatively what was anecdotally reported in media and case reports (Chen et al., 2025).

However, a few papers noted *pockets of satisfaction*. For example, (Hunt and Samman 2020) found that a subset of gig workers appreciates not having a boss, even if the algorithm is demanding—they preferred impersonal algorithmic feedback to potentially biased human supervision. Moreover, in a rare positive framing, a study by (Wiener et al. 2023) discussed how some drivers perceive the algorithmic system as providing a sense of “*legitimacy*” or fairness, primarily when it protects them from demanding customers by enforcing rules on both sides. This underlines that not all workers uniformly hate algorithmic management; perceptions can vary based on individual expectations and alternative job comparisons.

#### 4.1.3 Worker wellbeing and psychological impacts

The literature decisively links algorithmic management to various strains on worker wellbeing. We have substantial evidence of stress, burnout, and dissatisfaction. As noted, (Zhang C. et al. 2025) explicitly measured increased burnout associated with algorithmic control through an ego-depletion mediator (Wang et al., 2024). Another survey by (Dong et al. 2025) on ride-share drivers indicated high levels of emotional exhaustion and depersonalization (hallmarks of burnout), which the authors attributed to the combination of monotonous tasks and constant surveillance. Many gig workers face income volatility due to algorithmic scheduling and pay fluctuations. Papers such as “Income Volatility in Gig Work” (Farrell and Greig, 2016; Hall and Krueger, 2018) have quantified significant weekly volatility in earnings. This financial stress is part of the overall impact on wellbeing. Chen et al.'s (2025) study focusing on safety performance is notable: it not only recounts accidents but finds algorithmic features like “*tracking evaluation*” and “*behavioral constraints*” had adverse effects on safety (through ego-depletion increasing risky behavior). This suggests a concrete health impact: algorithmic management can indirectly contribute to workplace accidents and physical harm. Although less studied quantitatively, qualitative research highlights anxiety, uncertainty, and dependence among gig workers, particularly in relation to opaque evaluation systems and unpredictable performance criteria (Rahman, 2021).

Yet, some coping is reported: workers deriving “*meaning*” by reframing their work (e.g., seeing themselves as entrepreneurs or enjoying the customer interaction more than the algorithmic aspects). (Vogel et al. 2020) discussed “meaningfulness misfit”—when gig workers' need for meaningful work is not met, it affects engagement. Gig work often scores low on meaningfulness due to its fragmented nature, which is an indirect indictment of algorithmic micro-management reducing tasks to rote actions.

#### 4.1.4 Resistance and collective action

The literature documents a rich array of worker resistance strategies, as earlier described. (Tassinari and Maccarrone 2020) analyzed food couriers' protests in Italy and the UK as new forms of labor contention, highlighting how technology both hampered and helped organizing. They found that even without an employment status, workers used social media to coordinate “strikes” (e.g., logging off en masse), which leveraged the algorithm against the platform, as the shortage of drivers would trigger surge pay—a tactic of forcing concessions.

(McDaid and Free 2025) provide a detailed account of online forums enabling resistance. Their identification of three mechanisms (solidarity, information exchange, discursive justifications) shows how a new digital collective repertoire is forming. This is a significant contribution, as it suggests that algorithmic management, despite physically isolating workers, has inadvertently fostered digital communities of workers, potentially sowing the seeds for a collective identity akin to a “digital union hall.”

On formal organizing, some encouraging findings. There are instances where legal victories (e.g., the UK Supreme Court Uber case) were precipitated by long efforts of driver collectives and unions (UK Supreme Court, 2021) showing that persistence can yield structural change (Uber had to implement minimum wage and holiday pay in the UK for drivers as “workers” after that case, albeit the algorithm remains in use, it had to adjust payouts).

However, some literature is skeptical about the sustainability of resistance. A piece by (van Doorn 2017) argued that while there are bursts of collective action, the disjointed nature of gig work makes long-term organizing difficult, and platforms adapt (e.g., Uber has given small perks to drivers or improved communication after strikes to diffuse momentum).

#### 4.1.5 Global and regional insights

A clear geographical imbalance characterizes the existing body of research on algorithmic management. As illustrated in Figure 4, Europe accounts for the largest share of empirical studies (38 of 103, or roughly 37% of the corpus). Much of this work centers on labor process theory, worker resistance, and regulatory experiments in the UK, Germany, Italy, and other Western European contexts. North America follows with 22 studies (about 22%), dominated by research on Uber, Lyft, and Amazon Mechanical Turk, which laid much of the conceptual foundation for understanding gig work and algorithmic control.

Asia contributes 19 studies (approximately 19%), reflecting growing scholarly engagement, particularly from China, South Korea, and India. Recent publications by authors such as (Wang et al. 2024), (Li et al. 2025), and (Lata 2025) signal the emergence of a regional research base examining platform labor in rapidly digitizing economies. Cross-national or global studies comprise 15 publications (15%), many of which are led by institutions such as the ILO or the World Bank. In contrast, Africa (seven studies, 7%) and Latin America and the Caribbean (two studies, 2%) remain significantly underrepresented. This persistent imbalance underscores a significant research gap: while the rhetoric of “global platforms” prevails, most empirical knowledge continues to originate from and circulate within the Global North.

From the studies that do exist in Africa, it was found that in Ghana, Kenya, Nigeria, and South Africa, ride-hail drivers face even more precarious conditions (earnings often below minimum wage) but have started to create new alliances (e.g., independent driver unions collaborating with traditional unions) (Akorsu et al., 2022; Ekdale and Aidoo, 2024; Hunt and Samman, 2020). They identified a regulatory vacuum as a significant issue—e.g., Nigeria drafting rules but not finalizing them, leaving algorithms completely unchecked by labor standards. The concept of “voice and representation” is key; it examines whether algorithmic work can be a catalyst for trade union revitalization by compelling unions to innovate. Their findings are cautiously optimistic about some local successes (like union recognition of platform issues) but overall depict a struggle. In Asia, research [e.g., by (Aneesh 2009) or others] has noted differences like the presence of local competitors such as Ola, which sometimes forces Uber to alter algorithms (like adding a cash payment option). Also, culturally, the prevalence of informal labor means gig work may be perceived differently. Some studies mentioned how Indian and Bangladeshi ride-share drivers use informal networks (like older taxi unions influencing new drivers) (Lata, 2025). One interesting case (e.g., Chen et al., 2025) offers insights, such as the Taiyuan accident story, which led to government action. Those papers illustrate that in China, state intervention can be swift once an issue is recognized, e.g., ordering platforms to ensure reasonable delivery times. This contrasts with the initially more laissez-faire approach in the US. Many European studies emphasize institutional context—how algorithmic management is tempered or contested by European work councils, or local court rulings.

For example, an article by (De Stefano 2018) noted that even if algorithms manage, EU courts might interpret heavy algorithmic control as evidence of employment (the logic used in some decisions). Ironically, algorithmic stringency could backfire legally in contexts with strong worker protection, a point raised by scholars like (Prassl 2018). The corpus had limited data, but I know from other sources that countries like Brazil see high adoption of platforms. A Latin American report by the ILO (2025b) suggests that workers are forced to work longer hours and use multiple apps to earn a sufficient income due to lower fare algorithms. Worker protests have also occurred (e.g., Brazil's 2021 “Breque dos Apps” strike).

These variations emphasize that local socio-legal frameworks have a significant influence on algorithmic management.

#### 4.1.6 Legal and regulatory developments

Many studies include discussions of the ongoing legal battles and policy measures, which I touched on. Court cases [e.g., employee status lawsuits (Uber BV v Aslam)] (UK Supreme Court, 2021) are frequently cited. Some papers analyze their implications; for example, one source notes the continual legal challenges in the on-demand economy, which blur the lines between contractor and employee. (Dubal 2020) is cited regarding how framing workers as entrepreneurs vs. employees is an ideological battle. A trend in literature from 2016 onward, more articles incorporate mention of “regulatory responses” or have sections on “Implications for regulators.” This reflects the maturation of the discourse, shifting from merely describing phenomena to influencing policy and practice.

The SLR findings confirm much of what I outlined in the literature review narrative, with the added weight of multiple sources supporting each point. The effects of algorithmic management are primarily seen as problematic for workers, reducing autonomy, increasing precarity and stress, and challenging existing labor frameworks, while offering some efficiencies and flexibilities that need to be reconciled with fairness. The research gap in global understanding is notable, as is the shift toward addressing these issues through policy and collective action.

I now proceed to the second part: the CDA findings, which reveal how these issues are framed and discussed in the literature and beyond.

### 4.2 Discursive interpretation of the identified themes (CDA)

The CDA uncovered several notable discursive patterns in how algorithmic management in the gig economy is discussed in academic and policy circles. These patterns illuminate the underlying values, assumptions, and contested ideologies in this domain. I presented the findings in terms of dominant discourses or “frames,” along with illustrative examples drawn from text analysis.

#### 4.2.1 Techno-optimist/managerial discourse

This frame is characterized by a neutral to positive tone regarding algorithmic management, often focusing on efficiency, innovation, and managerial improvements. It is commonly found in information systems and management literature, as well as in corporate or industry communications. In this discourse. Terminology often leans toward words like “innovation,” “optimization,” “efficiency,” and “flexibility,” and frequently employs corporate language (see Duggan et al., 2023; Lee et al., 2015; Meijerink et al., 2021; Waldkirch et al., 2021). Gig workers might be referred to as “service providers” or “independent contractors” rather than employees or workers, aligning with platform-preferred terms. For instance, an HRM-oriented article described algorithmic HRM as an “*omniscient control system*” that “*differs from traditional control in its reliance on technology*,” a somewhat value-neutral phrasing that sidesteps emotive terms (Duggan et al., 2023). This discourse often implicitly adopts the perspective of the platform or the efficiency seeker. Workers are sometimes portrayed as factors in a system to be managed. (Wiener et al. 2023) framed the issue as a “legitimacy challenge” for platforms, focusing on how platforms can tweak their algorithms to gain driver trust. This framing assumes the continuation of algorithmic control and seeks ways to make it palatable, rather than questioning it. For example, “*Algorithmic management can enhance consistency in HR decisions and reduce human bias*”—such a statement, which I saw paraphrased in several works (Patil, 2025; Zhu G. et al., 2024), indicates an optimism that algorithms are improvements if properly implemented. The use of “enhance” and “reduce bias” casts algorithms as solutions. This discourse often omits direct discussion of power or exploitation. It rarely uses words like “precarious” or “unfair.” If worker issues are mentioned, they are referred to as “challenges” or “concerns” rather than systemic critiques (e.g., “*one challenge is maintaining worker engagement under automated systems*”—a sanitized way to discuss potential discontent).

The effect of the techno-optimist discourse is to normalize algorithmic management as a natural evolution. It aligns with a broader narrative that more data and automation are inherently beneficial. This discourse can be influential in policy because it often surfaces in white papers or expert consultations where the focus is on “*harnessing technology for growth*.” I observed, for example, in some policy-oriented documents (European Parliament, 2025; HRW, 2025; ILO, 2025a), a phrasing like “*platform work offers flexibility and opportunity, but we must update regulations*,” which starts positive before acknowledging issues—indicating an optimism-first approach.

#### 4.2.2 Critical labor discourse

In contrast, a strong critical discourse is present in sociology, geography, law, and critical management studies. This discourse explicitly highlights themes of exploitation, surveillance, and inequality. It frequently uses charged terms like “*exploitation*,” “*precarity*,” “*surveillance*,” and “*control*.” Metaphors are invoked: “*digital Taylorism*,” “*platform capitalism*,” “*the algorithmic panopticon*,” “*Wild West*” (for unregulated context), “*dark side of automation*,” etc (Alacovska et al., 2024; Gandini, 2019; Lata, 2025; McDaid and Free, 2025; Rosenblat and Stark, 2016; Wood et al., 2019a,b; Zuboff, 2023). For instance, (Cameron 2024) used the phrase “*retreat from direct control*,” referencing Vallas and Schor, to discuss how algorithmic management hides control behind technology. Workers are referred to straightforwardly as “workers” (or sometimes “labor” or even “laboring bodies”), emphasizing their status as laborers deserving rights. Platforms might be referred to as “employers in denial” or “lean platforms,” stressing the critique that they are employers avoiding responsibility. This discourse does not treat algorithms as autonomous; it often brings back human or institutional agency, e.g., “*Uber uses algorithms to exert control while circumventing labor laws*.” The frequent reference to company names (Uber, Deliveroo, etc.) in critical pieces attributes responsibility and counters the notion that “the algorithm” did it by itself. A law scholar like Dubal might write, “*Algorithmic control evades legal accountability by hiding behind the facade of neutral technology, thus exacerbating the wage slave vs. entrepreneur false dichotomy*” (Dubal, 2017a). Here, “evades” and “facade” signal intentionality and deception; “wage slave” is a highly charged term historically, as used in Dubal's work. Similarly, phrases like “*fissured workplace*” or “*taking the boss out of the equation but keeping the boss's power*” appear in this discourse. Many of these texts conclude with calls to action (e.g., the need for stronger regulation, adaptation to collective bargaining, etc.). They treat algorithmic management as a problem to be solved or, at the very least, tamed, rather than as a neutral phenomenon.

This critical labor discourse empowers the perspective of workers and is often aligned with advocacy. It provides intellectual backing to labor movements and has penetrated media: journalists usually use similar framing now, talking about “exploited gig workers” referencing academic studies. However, one observed risk is that this discourse can sometimes be *dismissed as alarmist* by the opposing side; thus, the discourse itself becomes part of the struggle. I noticed, for example, that Uber's own statements often implicitly refute the critical discourse without naming it (they will say “our drivers love the flexibility,” countering the claim of exploitation, etc.).

#### 4.2.3 Legal-technical discourse

This is found in law reviews and policy analysis. It is somewhat intermediate in tone, that is, serious and concerned with definitions and evidence, but less emotive than the labor discourse. It uses formal legal terms: “*worker classification*,” “*liability*,” “*due process*,” “*antidiscrimination law*,” etc. For example, “*the on-demand economy blurs the independent contractor–employee boundary*” (Cameron, 2024) is a matter-of-fact statement in a legal article. I see phrases like “*dualism of legal worker identities*” (Dubal, 2017b) or “*triadic relationship*” in some legal analyses, framing it in academic terms of labor law theory (Adams-Prassl et al., 2023). Terms such as “decent work,” “social protection,” and “policy vacuum” are used. For instance, some EU policy documents mention ensuring “*decent working conditions in platform work*” (European Parliament, 2025; Fredman et al., 2025). Often, these texts acknowledge both sides, noting the benefits of platforms while stressing the need for modernization of the law. They might say, “*while platform work provides innovation, it challenges existing legal frameworks designed for traditional employment*”—a balanced framing which then usually leads to suggesting legal reforms (like clarifying employment status or mandating transparency).

This discourse can be persuasive to policymakers because it is presented in a rational and evidence-based tone. It often draws on data (such as percentages of workers below the minimum wage, etc.). It positions itself as finding solutions (e.g., proposing new legal tests or bargaining frameworks).

##### 4.2.3.1 Worker narrative in qualitative accounts

Through CDA, I also got glimpses of workers' own voices as reported in qualitative studies and forums. This is not a published discourse in the same sense, but it appears in quotes and ethnographic descriptions. A mix of resignation, humor, and anger in how workers describe algorithms. They often anthropomorphize, referring to the app as “Big Brother” or “the boss” (Rosenblat and Stark, 2016). One driver, quoted in (Cant 2019, p. 35), stated: “*I think of the app as my boss – a boss that's a jerk sometimes*.” Another driver quoted in Rosenblat and Stark's (2016) study said, ‘she tells me where to go,' ‘she's moody,' ‘she hides jobs' using human metaphors for opaque algorithmic behavior (Rosenblat and Stark, 2016; Tassinari and Maccarrone, 2020; van Doorn, 2017). Such quotes demonstrate workers making sense of the algorithm by equating it to a human manager, revealing the fundamental power dynamic it holds for them. Storytelling is a common phenomenon: for example, drivers sharing cautionary tales of being unfairly deactivated; these serve as folk discourse that spreads in forums, shaping a collective understanding that “*the algorithm is not your friend*.” Interestingly, some workers adopt company rhetoric when it suits them (e.g., “I like being my own boss”), while also complaining about specific features (“the rating system is BS, one unruly pax [passenger] can ruin you”). Workers thus navigate between the flexibility frame and the exploitation frame in their own talk. Some workers internalize blame (e.g., “if I was deactivated, maybe I didn't keep my ratings up”), showing how the discourse of performance is internalized. In contrast, others collectivize blame, arguing that “Uber's algorithm is unfair, we should demand change.”

The inclusion of these voices in academic discourse (through quotes) sometimes challenges more sanitized academic language, bringing raw experience into the discourse. It grounds the critical claims in human stories, which is a decisive rhetorical move.

#### 4.2.4 Use of metaphors and historical analogies

I found the discourse frequently draws analogies to past labor systems, “Taylorism/Fordism.” Already mentioned, framing gig work as a new Fordist assembly line distributed via apps (Cant, 2019; Moore M. G., 2018; Moore P. V., 2018; Wood et al., 2019a). For example, some refer to gig work as “*just-in-time labor, analogous to just-in-time manufacturing*” (De Stefano, 2016b), highlighting the continuity with old productivity techniques. “Panopticon,” the concept of omniscient surveillance from Bentham/Foucault, is directly referenced in some discourses (Newlands, 2021; Woodcock, 2020), indicating scholarly attempts to frame the phenomenon in well-known theoretical terms related to surveillance. I saw references to call center literature (Bain and Taylor, 2000) using terms like “electronic panopticon” and more contemporary works extending that to gig (some have called Amazon's Mechanical Turk a “virtual sweatshop”) (Pittman and Sheehan, 2016). Another metaphor that appears is that algorithmic management is “a game” or uses “gambling” tactics, such as variable rewards (some liken driver incentives to a slot machine effect—unpredictable rewards that hook behavior).

Metaphors matter because they make complex issues accessible and shape opinion. “Digital Taylorism,” for instance, clearly connotes exploitation and lack of creativity, potentially swaying opinion against the gig model if it sticks. Meanwhile, platform advocates avoid these metaphors, instead preferring “network” or “marketplace” metaphors (trying to frame gig workers as like small business owners in a marketplace). That is another discursive clash: *market metaphors* vs. *factory metaphors*. The market implies voluntary exchange among equals; a factory implies hierarchy and exploitation. This analysis found that labor-critical discourse uses factory metaphors (“gig workers on an assembly line of code”). In contrast, platform discourse uses market metaphors (“we are just connecting supply and demand”).

#### 4.2.5 Power dynamics in discourse

Finally, the CDA reflected on how discourse itself is a site of power. Platforms have tried to control the narrative (Uber's PR campaigns about driver flexibility, Prop 22 ads featuring drivers who say “I love being independent”). There is a *corporate discourse* that has not been thoroughly analyzed in our text-based approach, due to the focus on academic sources, but is evident in the media—it promotes the notion that “gig work is empowering entrepreneurship” (Dubal, 2017a; Rosenblat, 2018). This directly conflicts with academic critical discourse. Academics and worker advocates have been gradually influencing media and policy discourse. For example, the widespread use of the term “algorithmic accountability” in policy discussions (European Commission, 2020; OECD, 2022) shows academic influence. Terms like “precarious” to describe gig jobs are now featured in mainstream news, reflecting the critical discourse's penetration. There is also a geographical power in discourse: much of published discourse originates in the Global North, possibly marginalizing how gig work is framed in Global South contexts. For example, the concept of “algorithmic management” might not be phrased in this way in local discussions elsewhere; they might simply refer to “app rules,” but global discourse (mainly in English) frames it in specific theoretical terms. This can shape which issues receive attention (perhaps bias and privacy receive much attention, which are Western concerns, vs. raw income security, which might be a more immediate concern in poorer countries).

This analysis suggests that to be truly global and just, the discourse should include perspectives and voices from the Global South more prominently and perhaps adjust some terminology accordingly. The CDA reveals a contested discursive field. On one side, a narrative of innovation and personal freedom. On the other hand, a narrative of exploitation and an urgent need for intervention. Legal/policy discourse tries to mediate between these, seeking a balance. Worker narratives add authenticity and urgency to the latter critical side, though not always directly heard in policy halls.

Understanding these discourses is crucial because they shape the *solutions that are considered*. For instance, if the dominant discourse in a country is “gig work = entrepreneurship,” policies will favor minimal regulation. If it is “gig work = exploitation,” we get stronger labor rights responses. The findings, therefore, highlight *how our approach to discussing algorithmic management influences our actions*. Having presented both the empirical/thematic findings, as well as the discourse analysis, I now move to discuss the implications of these findings, synthesizing them to identify the novel research gap and contributions, and exploring the broader implications for theory, practice, and policy.

## 5 Discussion

This review and analysis illuminate the multifaceted nature of algorithmic management in the gig economy, bridging empirical evidence with critical interpretation. Below, I discussed the implications of the findings in light of the research questions, highlight the novel research gap identified, propose an integrated research agenda, and elaborate on the broader legal, ethical, social, and policy implications. I also reflect on the study's limitations.

### 5.1 Addressing the research questions and gaps

#### 5.1.1 RQ1 (Key themes and findings across global regions)

The SLR distilled several core themes: widespread algorithmic control mechanisms, reduced worker autonomy, significant stress and precarity for workers, emergent forms of resistance, and diverse regional manifestations. While these themes resonate with earlier understandings of gig work, the comprehensive approach confirms their validity across a vast body of literature and updates them with recent studies (up to 2025). A crucial insight is that algorithmic management consistently tilts the balance of power in favor of platforms by enabling granular control over a distributed workforce (Kellogg et al., 2020; Wood et al., 2019a). This holds globally, yet the magnitude of impact varies by context: where labor protections are strong (e.g., parts of Europe), some excesses of algorithmic control are curtailed or moderated, whereas in more deregulated contexts (e.g., many developing countries), algorithmic control can operate unabated, often leading to even harsher conditions (longer hours for less pay, greater safety risks, etc.) (Anwar and Graham, 2021; ILO, 2021a; World Bank, 2023). I found that scholarly attention is now expanding to these under-represented regions, but still lags, marking a research gap in cross-regional comparative studies.

One novel pattern that emerges is the concept of “contextual algorithmic management”—algorithms may be global, but their effects are local. For example, drivers in Nairobi or New Delhi might experience the same Uber app as those in New York. However, their earnings, social status, and recourse options differ vastly, shaping a different reality of algorithmic management. The literature hints at such differences (Ekdale and Aidoo, 2024; Sehrawat et al., 2021), but comprehensive comparative frameworks are lacking. I identify this as an impactful gap: future research should systematically compare how algorithmic management outcomes differ in the Global South vs. the North, or under different regulatory regimes, while controlling for platform and task type. Such research could develop a more nuanced theory that incorporates institutional and cultural moderators into the effects of algorithmic management (some initial theoretical work by Rosenblat and Stark, and others are steps in this direction, but empirical follow-through is needed).

#### 5.1.2 RQ2 (Discursive framing and patterns)

The CDA revealed that discussions of algorithmic management are not value-neutral; they are imbued with ideological underpinnings. I observed a polarization of narratives—with platform- and tech-friendly narratives emphasizing flexibility and innovation, and labor-critical narratives emphasizing exploitation and surveillance. This polarization matters because it can lead to talking past each other in public debate and policy discourse. For instance, if a government hearing on gig work features gig company representatives vs. labor advocates, they might present completely different pictures of the same phenomenon: one citing the number of people earning “on their own terms,” and the other citing instances of hardship and unfair terminations (ILO, 2021a). This analysis suggests that bridging this gap requires reframing the conversation. Some scholars are indeed attempting a reframing: introducing concepts like “algorithmic dignity” or “fairwork” (as seen in the Fairwork project's discourse) that try to capture worker wellbeing without rejecting the digital innovation outright.

An important discursive finding is the role of metaphor and historical analogy—referring to algorithmic management as “digital Taylorism” (Moore M. G., 2018; Rosenblat and Stark, 2016) or “a new form of piecework”-situates it historically and normatively (usually negatively, as those terms carry baggage). These analogies can be powerful rallying calls (e.g., for unions to view gig work as just another form of exploitative labor that needs organization). On the other hand, the platform narrative equating gig work with entrepreneurship aligns with neoliberal ideals of self-reliance, which can garner public sympathy, especially in cultures that celebrate entrepreneurship. The tension between these frames suggests that the outcome of labor reforms may partly depend on which narrative gains prominence. This CDA contributes by making these frames explicit, hopefully aiding scholars and activists to be more strategic in communication. It also encourages reflection within academia: are researchers inadvertently biased toward one frame? For example, managerial journals that use specific terms might unknowingly perpetuate a discourse that sidelines worker hardships (by, for instance, always referring to them as “contractors” rather than “workers”). As academics engaged in this topic, being conscious of language is a takeaway; hence, ensuring we clarify terms and perhaps adopt more neutral or worker-inclusive language where appropriate (e.g., using “gig worker” instead of “service provider”) could be a small but meaningful shift in scholarly writing.

#### 5.1.3 RQ3 (Gaps and practical concerns)

The gap I underline is comparative global research with contextual sensitivity, as discussed. Another gap is longitudinal research; we have many cross-sectional snapshots of gig conditions, but few studies track how algorithmic management and worker responses evolve over time. Will workers adapt more and get better at resisting? Will algorithms become even more advanced (with AI) and possibly address some current complaints or create new ones? The literature lacks a temporal dimension; almost all studies treat the early 2020s situation as static. I see an opportunity for longitudinal ethnographies or panel surveys that follow the same gig workers through changes (like when Uber changes an algorithmic policy, what is the effect?). Additionally, as suggested by the findings, as more companies outside the gig economy adopt algorithmic management tools (e.g., algorithmic scheduling in retail stores and warehouse work monitored by AI cameras), a gap remains in exploring commonalities between the gig and traditional sectors. This could lead to a broader understanding of algorithmic management as not just a gig phenomenon, but a workforce-wide trend. Some included sources (e.g., Moore and Joyce, 2020) suggest that line, but more research can connect gig scholars with those studying AI in traditional workplaces.

From a practical standpoint, the findings raise concerns that call for action:

#### 5.1.4 Legal concern

The persistent misalignment between gig work realities and legal categories, if not resolved, threatens to leave millions of workers in a protection limbo. This is a legal ticking clock; some jurisdictions are moving (EU), others are stuck. This review supports the argument (Dubal, 2017b, 2020; Vallas and Schor, 2020) that maintaining the status quo perpetuates injustice. Therefore, there is an imperative for legal innovation, whether that involves expanding definitions of employment or creating a third status with meaningful protections (although this is a controversial approach); something must likely change. The study's comprehensive evidence can inform such legal reforms by highlighting exactly which aspects of algorithmic management are most harmful (e.g., a lack of transparency in firing decisions).

#### 5.1.5 Ethical concern

Ethically, platform companies have a responsibility to do better. Research indicates that minor adjustments (e.g., providing couriers with slightly more delivery time or a “pause” button to take breaks) can have a significant impact on safety and stress (ILO, 2021a; Tassinari and Maccarrone, 2020). Companies often know these issues (some internal documents have leaked, showing awareness). However, the incentive to maximize efficiency holds them back. However, sustained public and regulatory pressure—which the discourse analysis indicates is growing—might push a more ethical stance. Some platforms have started pilot programs (like allowing tipping or providing more info to workers about how pay is calculated after public criticism). The findings bolster the ethical case: ignoring worker wellbeing not only harms workers but can also backfire on platforms through negative publicity, legal challenges, and even reduced service quality (burnt-out drivers often provide poor service).

#### 5.1.6 Social concern

On a societal level, a risk emerges that algorithmic management could deepen socio-economic inequalities. Already, certain marginalized groups may self-select into or be funneled into gig work; if algorithms have biases (even subtle ones, such as geographic allocation that might favor drivers from affluent areas), disparities can grow. Additionally, suppose we accept that a portion of the workforce will be subject to algorithmic control. What implications does this have for democratic values in the workplace? There is a cultural question: if people become conditioned to being managed by opaque systems, it might alter expectations of autonomy in other spheres. This is more speculative, but some sociologists warn about the normalization of surveillance and rating systems beyond work (as in “reputation economy” ideas). The review did not deeply delve into non-work contexts, but it is a connected social issue.

Looking forward, this interdisciplinary framework and its findings suggest that a conceptual shift is necessary in how we approach algorithmic management. Instead of viewing it solely as a tech-driven efficiency measure, it should be recognized as a form of governance or rule-making that needs legitimacy [as some authors have argued (Wiener et al., 2023)]. If we treat platforms as governors of work, then principles of good governance (transparency, accountability, participation) should apply. This shift aligns with the discourse of “democratizing the algorithm,” giving workers a voice in algorithm design or rules. It might sound far-fetched, but there are nascent efforts (the course of cooperative platforms, or initiatives where workers negotiate changes in app features, as seen in some Deliveroo meet-ups with rider representatives). The review encourages considering algorithmic management as not set in stone; humans design it and can be redesigned with more stakeholder input.

### 5.2 Legal, ethical, social, and policy implications

I have touched on many implications, but to compile them clearly:

#### 5.2.1 Legal implications

There is increasing legal recognition that algorithmic management can make a company an employer in all but name (Aloisi and Gramano, 2019; Cherry, 2021; Prassl, 2018). Laws and courts are beginning to catch up, but their application is inconsistent across jurisdictions (see: *Uber BV v Aslam*). This review implies that jurisdictions that proactively clarify worker status and rights will better protect workers and potentially foster more sustainable platform models (since ongoing legal uncertainty also hurts business in the long run). Additionally, labor law may require new provisions specific to algorithmic management—for example, requiring companies to disclose the criteria for deactivation and allow for appeal (essentially, adding due process rights in the labor law context for algorithm-driven decisions) (Aloisi and De Stefano, 2024). Anti-discrimination law might also need updates: if an algorithm's pattern of work assignment disadvantages a group (say, fewer gigs for those in specific neighborhoods), is that discrimination? Regulators should consider algorithmic bias under existing civil rights laws (some proposals call for treating algorithms as “agents” of the company, thus the company is liable for their decisions).

#### 5.2.2 Ethical implications

Ethically, the findings necessitate that stakeholders treat gig workers as human beings deserving of dignity, rather than as units of flexible labor. This moral standpoint would insist on things like no worker should earn below a living wage under an algorithm's regime (the ethics of a fair wage), workers should have the right to know why a decision was made about them (the ethics of transparency and respect), and algorithms should incorporate safety margins to not push humans beyond safe limits (the ethics of care). The mention in my sources that “*exhausted riders meet unrealistic deadlines*” “*riders being forced to rush to meet algorithmic delivery times, often skipping meals or breaks, resulting in chronic fatigue and near misses*” (Dong et al., 2025; ILO, 2021b) is an ethical red flag (Floridi, 2019; Jobin et al., 2019)—it contravenes the obligation not to put workers in harm's way. So, ethically, platform companies must re-evaluate their algorithms under a duty of care framework. Additionally, considering the broad impact, allowing a segment of the workforce to be managed by inscrutable algorithms might be seen as treating them as second-class workers. An ethical society should strive to establish standards for gig work that align with those of traditional employment in terms of respect and rights.

#### 5.2.3 Social implications

At a societal level, one implication is the potential erosion of the social contract between workers and firms. Gig work and algorithmic management externalize risk onto workers, as many authors note (HRW, 2025; Li et al., 2025; Mbare et al., 2024; van Zoonen et al., 2025). If this model expands unchecked, more workers will live without the security nets and reciprocal loyalty that characterized mid-20th-century employment, which can lead to social instability (people unable to plan futures, get loans, etc., due to income volatility). Some see a danger of a fragmented society where a class of workers is algorithmically managed with precarity (the “precariat”), which could have political ramifications (anger, populism, etc.). On the positive side, some argue that gig platforms could uplift people by providing access to work where jobs are scarce (e.g., rural areas or developing countries). This review suggests that the algorithm's operation limits the benefit. Suppose it floods the market with too many workers (which many platforms do to ensure availability, leading to an insufficient number of gigs per person). In that case, it may not actually alleviate poverty much but instead spread thin earnings among many. It is a complex social calculation. Regardless, society must grapple with whether we want “algorithmic bosses” as a norm. There is also the future-of-work aspect: as AI gets more sophisticated, algorithmic management might evolve to manage even complex work (some knowledge work jobs now have dashboards tracking performance). Society might need to set boundaries on acceptable uses of such management (like how the EU is considering banning specific AI uses that are too intrusive in workplaces).

### 5.3 Policy implications

Drawing on patterns identified across the reviewed literature; several policy directions emerge. Extending beyond existing literature, this review also proposes additional normative recommendations aimed at strengthening governance of algorithmic management in the platform economy. The recommendations below therefore combine (a) policy measures already discussed in existing scholarship and institutional reports; and (b) proposals advanced in this review to address gaps identified through the synthesis.

- Immediate protections: establish minimum wage floors for gig work and mandate contributions to social insurance (some countries are implementing this by requiring platforms to contribute to funds for gig workers).
- Algorithmic transparency laws: require platforms to disclose to workers the key factors of their algorithms and possibly audit algorithms for bias or unfair practices (the way credit scoring algorithms are somewhat regulated).
- Support collective representation: adapt labor law to allow gig workers to organize without violating antitrust (since currently they are considered independent businesses, collective organizing can be seen as price-fixing). Some jurisdictions have granted exemptions to allow collective bargaining for gig workers, a significant policy change that encourages this approach.
- Invest in technology for good: governments or public-private partnerships could develop open-source or ethical algorithmic management systems that cooperatives or fair platforms could adopt, demonstrating that gig work can be managed in a more equitable way (e.g., Fairbnb vs. Airbnb-type alternatives).
- International coordination: since platforms operate globally, having very different rules by country can create regulatory gaps. Extending beyond the specific policy measures identified in the literature, this review suggests that international labor standards for platform work may eventually become necessary, a possibility already being explored in discussions within institutions such as the ILO. This review, covering global evidence, could inform the development of such international guidelines.

### 5.4 Limitations and future research

No study is without limitations. Covering 103 sources in one article means we often summarize studies rather than providing fine-grained detail of each. There is a risk of oversimplification or missing nuances in specific contexts (for instance, gig work, such as ride-sharing, vs. domestic work via platforms, might have differences that we glossed over). Future research could delve into sub-sectors or specific regions through dedicated reviews. This review was limited to English-language publications. This linguistic restriction likely contributes to the overrepresentation of scholarship originating from the Global North, where English-language academic publishing dominates. Consequently, locally grounded research produced in other languages including Mandarin, Spanish, Portuguese, or French may be underrepresented in the final corpus. This filtering may therefore obscure important regional insights from the Global South and should be considered when interpreting the geographical distribution of studies identified in this review.

I encourage more translation and cross-pollination, or bilingual scholars to conduct parallel reviews. The platform economy is evolving (especially post-pandemic, e.g., food delivery boomed). The review is current as of 2025, but new developments (like advancements in AI management or new laws in 2026) could shift things. Thus, the conclusions should be revisited as the environment changes. I attempted to mitigate this by incorporating the most recent studies and highlighting emerging trends (such as AI integration), but the rapid pace necessitates ongoing research. This discourse analysis, while systematic, still involves interpretation. Another researcher might categorize some discourses differently or pick up on nuances I did not. I addressed this with cross-checks, but CDA is inherently a bit subjective. I view this as a worthwhile trade-off for insight. However, readers should consider it interpretative rather than purely objective truth. Algorithmic management also appears in traditional companies now (e.g., Amazon warehouses). I primarily focused on gig/platform contexts, so future research may explore how these findings apply to non-gig settings. There might be differences when workers are employees but still algorithm-managed (some research shows they at least have more recourse to complain via HR if they are employees).

### 5.5 Future research directions

Building on my identified gaps: comparative case studies: e.g., compare Uber's algorithmic management in two countries with different regulations—what differs in worker outcomes? Or compare two platforms in the same region with other models (e.g., Uber vs. a worker cooperative platform) to see how management differs. Longitudinal studies: follow a set of workers over time to observe how they adapt or how their opinions of the platform change, particularly in response to significant algorithm changes or life events. Algorithm audits with worker input: interdisciplinary projects where computer scientists audit a platform algorithm for fairness or efficiency, while social scientists gauge worker perceptions of that algorithm. This can connect objective measures with subjective experiences. Mitigation experiments: if a platform or third party implements a change (such as providing more transparency or forming a worker council that regularly meets with management to adjust algorithm parameters), study the effect on outcomes, including worker satisfaction, retention, and service quality. This could provide an evidence base for what reforms make a difference. The role of customers: I did not talk much about customers, but they are part of the algorithmic management equation (through ratings and demands). Studying how customer behavior and expectations (perhaps shaped by platform design) add to pressure on workers would be helpful.

Additionally, educating customers (e.g., encouraging fair ratings and offering incentives for tipping) could be a lever; research could explore this dimension. Evolution of discourse: it could be interesting to analyze media and policy documents over time to see if the framing of gig work is shifting (maybe due to activism or scholarly influence). That could validate some of my observations in a more quantitative way (e.g., content analysis of news articles from 2015 vs. 2025 to see if terms like “exploitation” appear more now).

## 6 Conclusion

Algorithmic management in the global gig economy represents a profound reorganization of work, blending technology, data, and economics in ways that challenge traditional labor practices and protections. Through this SLR and CDA, I have shown that while algorithmic management offers efficiency and scale to platform businesses, it often does so at the expense of workers' autonomy, security, and dignity. From the Global North to emerging economies of the Global South, gig workers navigate a double-edged sword: enjoying flexibility and income opportunities on one side, yet facing an opaque, relentless digital boss on the other.

This article makes it clear that these challenges are not isolated or anecdotal; they are documented in hundreds of studies and experienced by millions of workers. I also highlighted a pivotal research and policy gap: the need to incorporate global and contextual perspectives to understand and address algorithmic management practices fully. What works to protect a gig driver in London may need adjustment in Lagos or Lahore; thus, regulators and scholars must learn from each other across borders.

Crucially, this analysis reminds us that how we frame the narrative of the gig economy can shape its future. Suppose we continue to valorize “hustle” and “flexibility” without acknowledging the embedded inequities. In that case, we risk normalizing a 21st-century digital Taylorism, where workers are managed by unaccountable code. Alternatively, by adopting a critical and human-centered discourse that emphasizes fairness, transparency, and rights, we can influence public opinion and policy toward building a more equitable gig economy. Encouragingly, we see growing collective action and regulatory interest as signs that change is possible. Gig workers are no longer invisible—their stories of algorithmic injustices are reaching courts, legislatures, and popular media.

This comprehensive examination yields several actionable insights. For scholars, I provided a foundation to push the frontier on this topic, urging more inclusive and longitudinal research. For policymakers and industry, I offered evidence-based recommendations to mitigate the adverse effects of algorithmic management, including ensuring fair compensation and algorithmic transparency, as well as involving workers in the design of the systems that govern them. For workers and advocates, I validated their experiences with academic rigor and underline the power of solidarity and voice in confronting algorithmic power.

The gig economy is often touted as the future of work. If so, we must ask: *What future do we want?* One where algorithms serve as tools to empower workers and businesses alike, or one where they operate as instruments of unchecked control and inequality? The answer depends on the actions we take now. By reimagining algorithmic management through the lenses of justice, accountability, and human agency, we can strive for a future of work that is not only innovative but also inclusive, fair, and equitable. I expect that this article contributes to that vital endeavor, providing both the knowledge and the critical perspective needed to inform change. The gig economy's algorithms may be written in code. However, the rules by which they operate and whom they ultimately serve remain very much in human hands. Let us use that responsibility wisely, to ensure that the future of work is one in which technology and humanity progress together, not at each other's expense.
